# Enzyme-cargo encapsulation peptides bind between tessellating tiles of the bacterial microcompartment shell

**DOI:** 10.1016/j.jbc.2024.107357

**Published:** 2024-05-10

**Authors:** Shuang Gu, Jack Bradley-Clarke, Ruth-Sarah Rose, Martin J. Warren, Richard W. Pickersgill

**Affiliations:** 1 School of Biological and Behavioral Sciences, Queen Mary University of London, London, UK; 2 School of Biosciences, University of Kent, Canterbury, Kent, UK

**Keywords:** bacterial metabolism, cell compartmentalization, enzyme catalysis, peptide interaction, protein self-assembly

## Abstract

Bacterial microcompartments are prokaryotic organelles comprising encapsulated enzymes within a thin protein shell. They facilitate metabolic processing including propanediol, choline, glycerol, and ethanolamine utilization, and they accelerate carbon fixation in cyanobacteria. Enzymes targeted to the inside of the microcompartment frequently possess a cargo-encapsulation peptide, but the site to which the peptide binds is unclear. We provide evidence that the encapsulation peptides bind to the hydrophobic groove formed between tessellating subunits of the shell proteins. *In silico* docking studies provide a compelling model of peptide binding to this prominent hydrophobic groove. This result is consistent with the now widely accepted view that the convex side of the shell oligomers faces the lumen of the microcompartment. The binding of the encapsulation peptide to the groove between tessellating shell protein tiles explains why it has been difficult to define the peptide binding site using other methods, provides a mechanism by which encapsulation-peptide bearing enzymes can promote shell assembly, and explains how the presence of cargo affects the size and shape of the bacterial microcompartment. This knowledge may be exploited in engineering microcompartments or disease prevention by hampering cargo encapsulation.

Bacterial microcompartments are prokaryotic organelles consisting of encapsulated enzymes within a thin protein shell. The first bacterial microcompartments, observed as polyhedral structures in electron micrographs, were the carboxysomes of cyanobacteria (1) which enhance carbon dioxide fixation *via* encapsulation of rubisco and carbonic anhydrase (2). Later, similar structures were observed in heterotrophs, but only when grown on the substrate of the microcompartment *e.g.*, ethanolamine or 1,2-propanediol (3). The majority of the bacterial microcompartments break down a metabolic substrate and are called metabolosomes. The bacterial microcompartment shell functions as a semipermeable membrane for substrates and products and segregates the encapsulated enzymes (4). A recent paper catalogs the increasing known diversity and ubiquity of bacterial microcompartments (5). The shell confines toxic and reactive intermediates and enhances catalysis by increasing the concentration of enzymes and substrates. Off-pathway reactions are minimized by the segregation of the enzymes within the microcompartment. Targeting of enzyme cargo to the lumen of the bacterial microcompartment is typically by a 15 to 20 amino-acid residue amphipathic α-helix that is connected to the N- or C-termini of the cargo protein *via* a flexible linker (6, 7).

In the Pdu microcompartment, the encapsulated enzymes convert 1,2-propanediol to propionaldehyde *via* a cobalamin-dependent catalytic mechanism catalyzed by PduCDE (Fig. 1*A*). The aldehyde is subsequently converted to propionyl-CoA by PduP (8) before the CoA is regenerated by PduL during the production of propionyl-phosphate (9). There is evidence that the aldehyde dehydrogenase (PduP) and the diol dehydratase (PduCDE) are within the lumen of the microcompartment (10, 11). The requirement for PduL to regenerate CoA for PduP would imply that it too is localized to the microcompartment lumen (12). Several other enzymes (PduGHOS and PduQ) are involved in regenerating the cobalamin and NAD^+^ cofactors. Recombinant production of bacterial microcompartments has shown that in the presence of encapsulation peptide-bearing metabolic enzymes, the microcompartments are larger compared to empty shells (11).

**Figure 1:**
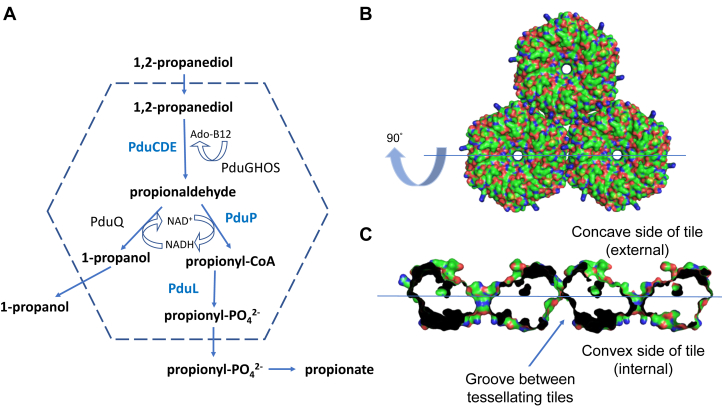
**The Pdu microcompartment and tessellating PduA hexamers which form the shell.***A*, schematic representation of the cobalamin dependent 1,2-propanediol utilization (Pdu) microcompartment and the encapsulated enzymes. Enzymes with known encapsulation sequences, PduCDE, PduL, and PduP, are highlighted in *blue*. *B*, three tessellating PduA shell protein hexamers viewed looking down on to the convex face of the hexamers. *C*, a central slab of the tessellating PduA molecules rotated about the horizontal axis by 90° and enlarged compared to panel (*B*) to show the concave and convex surfaces of the hexamers, the thin hexamer-hexamer interface can be clearly seen between tessellating hexamers. The hydrophobic groove formed between the tessellating PduA protein tiles is indicated on the convex side of the PduA hexamer. The three hexamers shown were generated using the crystal structure (Protein databank code: 3NGK) and the crystallographic symmetry.

The propanediol utilization (Pdu) metabolosome from *Salmonella* comprises eight shell proteins (PduA, B, B′, J, K, N, U, T) of which PduA, B, B′, J are major and PduK, T, U (and N) are minor components of the shell (13). The shell protein PduA consists of a single Pfam00936 domain that assembles into a cyclic homohexamer with a convex and concave side (Fig. 1, *B* and *C*) (14, 15). PduB is a tandem fusion of two Pfam00936 domains that assemble into a cyclic homotrimer which closely resembles the size and shape of the PduA hexamer (16, 17, 18). Except for the vertex-capping pentamer, PduN (19), the shell proteins are either hexamers or pseudo-hexamers. Several thousand of these hexamers and pseudo-hexamers tessellate to form the facets of the bacterial microcompartment.

In this work, we use the hexameric shell protein PduA from *Citrobacter freundii*. PduA has been shown to transport the substrate 1,2-propanediol to the lumen of the Pdu microcompartment (14). While PduA comprises only 19% of the shell proteins present in the microcompartment shell its sequence is closely homologous (80% sequence identity) to PduJ which accounts for 54% of the shell proteins (20). The structure of PduJ is nearly identical to PduA and the *pduA* gene complements the growth phenotype of a *pduJ* deletion mutant (21). Remarkably, given the substrate channel of PduJ is identical to that of PduA, it is the genomic position of the *pduJ* gene in the operon that determines its ability to act as a pore for 1,2-propanediol transport (21). Together then PduA and PduJ account for 73% of the tiles forming the microcompartment facet (20). We therefore argue that sheets of tessellated PduA hexamers and assembly intermediates involving PduA hexamers are reasonable proxies for the facet of the microcompartment.

Enzymes encapsulated within the Pdu microcompartment have a short, typically 15 to 20 residue, encapsulation sequence (22). These sequences form amphipathic helices (23) with small hydrophobic residues clustered on one side of the helix and are found at the N-terminus or C-terminus of the enzyme cargo (24). Within the Pdu metabolosome, the acylating propanol dehydrogenase, PduP has an 18-residue N-terminal sequence that facilitates encapsulation. Several computational methods have predicted that the encapsulation peptide of PduP binds to the concave surface of PduA, thus requiring the concave side of the PduA tile to be luminal (25, 26). However, there is now convincing evidence that the concave surface of the PduA tile is external both from structural studies of a recombinantly generated metabolosome (27) and from our own previous work (28). While there is some affinity for the concave (external) surface of the tile, the work we report here reveals the encapsulation peptide has a greater affinity for the hydrophobic groove on the internal convex side of the hexamer formed between tessellating tiles. Enzymes bearing encapsulation peptides are not essential for microcompartment assembly, but they do influence microcompartment assembly (11, 29). The binding of the encapsulation peptide between tessellating tiles therefore has important consequences for understanding the nucleation of bacterial microcompartment assembly in the presence of enzyme cargo and provides an explanation of how cargo influences microcompartment size and shape.

## Results

### PduA tessellation intermediates

**Figure 2:**
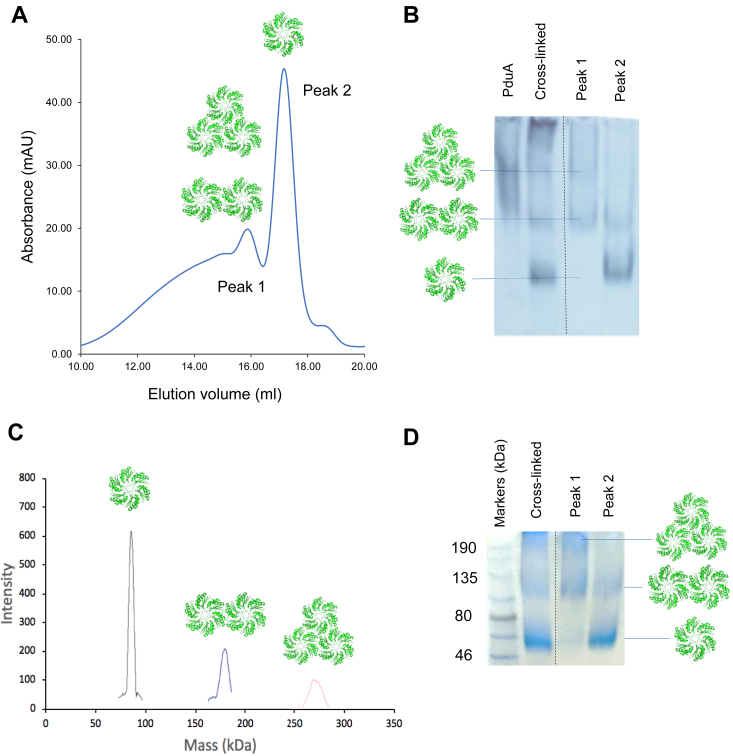
**Identifying early-stage tessellation intermediates.***A*, after sonication and cross-linking, low molecular mass oligomers of PduA hexamers can be separated using size-exclusion chromatography. The first peak corresponds to dimers and trimers of hexamers while the second peak is predominantly the PduA hexamer. *B*, the native gel is consistent with peak 2 comprising mostly monomer, peak 1 trimer and dimer. The glutaraldehyde cross-linked sample contains all three components, monomer, dimer, and trimer. The PduA samples used for the work described here were checked and were predominantly trimers of hexamers (first lane of gel). *C*, the mass of the peaks from the glutaraldehyde cross-linked sample was confirmed by MALDI-TOF mass-spectrometry. *D*, cross-linked samples run on SDS-PAGE gives molecular masses in agreement with the results of dynamic light scattering and mass-spectrometry. Some lanes were cropped from the gel images shown in panels (*B*) and (*D*) at the position indicated by the dotted lines.

PduA hexamers tessellate to produce protein sheets and nanotubes (15, 28). Substitution of key residues at the tessellation interface, lysine 26 and arginine 79, reduces the propensity of PduA to tesselate (15). Here, we use sonication to disrupt the sheets and nanotubes and produce assembly intermediates. Glutaraldehyde cross-linked samples of sonicated PduA were analyzed by size-exclusion chromatography and native gel electrophoresis (Fig. 2AB). The native gel from a typical cross-linked sample shows evidence for three distinct species in addition to higher molecular mass species too large to enter the gel (Fig. 2*B*). It is plausible that the three species are the PduA hexamer with the highest mobility, the dimer of hexamers, and the trimer of hexamers with the lowest mobility on the gel. Making this assumption then the two peaks from the size exclusion chromatography are mainly dimer and trimer of hexamers (Peak 1) and mainly monomer (Peak 2). Dynamic light scattering measurements from samples of peaks 1 and 2 have hydrodynamic radii of 6.68 nm and 3.71 nm, respectively. These values are not easy to replicate from simple calculations using the structures of the hexamer and trimer of hexamers suggesting there are some larger oligomers also present after cross-linking. Mass-spectrometry of cross-linked PduA revealed masses of 88 kDa, 176 kDa, and 264 kDa (Fig. 2*C*) which is consistent with a ladder of one, two, and three assembling oligomers although the cross-linked hexamer is presenting at higher mass than the PduA hexamer mass of 64 kDa. SDS-PAGE of the glutaraldehyde-modified PduA gives bands at approximately 60 kDa, 120 kDa, and 190 kDa (Fig. 2*D*) which is closer to multiples of the PduA hexamer (64 kDa). It is plausible the smaller oligomers that enter the native gel are hexamer, dimer of hexamers, and trimer of hexamers. The cyclic trimer of hexamers is shown in the cartoon in Figure 2 because the cyclic trimer is stabilized by three interfaces rather than only two interfaces in the linear arrangement. PduA without cross-linking tends to assemble into a trimer of hexamers although the band always smears suggesting there is heterogeneity in assembly present (Fig. 2*B*).

### PduL binds to tessellating PduA

**Figure 3:**
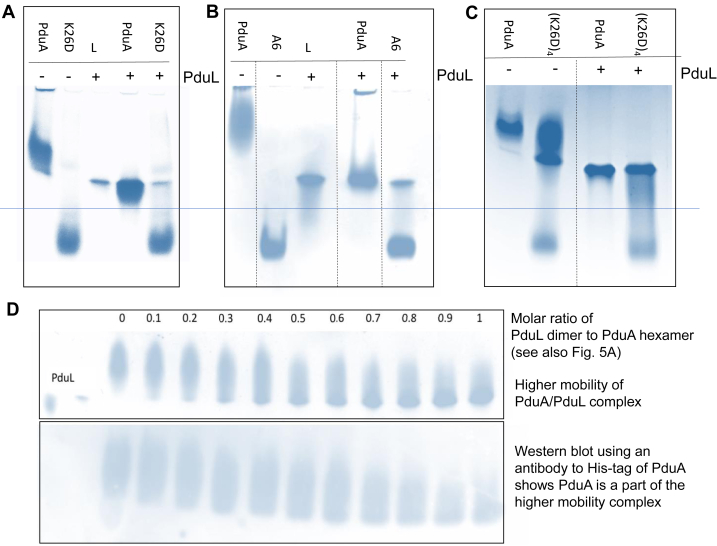
**PduL binds only to tessellating PduA hexamers.** Tessellating PduA has lower mobility on native PAGE and is observed above the horizontal blue line on the figure and the band shifts when PduL is added, non-tessellating PduA has higher mobility and is seen below the blue line. *A*, PduA and PduA variants without and with the addition of equimolar PduL (molarity calculated on the basis of the PduA hexamer and PduL dimer). The non-tessellating variant, K26D with the interfacial lysine 26 replaced by aspartate, has a lower mass and correspondingly higher mobility. The addition of the acidic PduL dimer has a profound influence on the mobility of the PduA trimer of hexamers, but not on that of the K26D mutant, where both PduA and PduL bands are unchanged. *B*, a similar result to that presented in the first panel is seen when the concatenated A6 PduA variant is used. This PduA variant does not tessellate and comprises six fused subunits in a single polypeptide chain, the individual subunits joined by linkers. Two distinct bands corresponding to non-interacting PduA variant A6 and PduL are seen indicating no binding. *C*, the (K26D)_4_ mutant of PduA, in which four of the six lysine 26’s are replaced by aspartate, tessellates slowly, over several days. Here is a sample that has been left for 2 days (lane 2). Some of the protein has tessellated (low mobility), some is still non-tessellating (high mobility). What is striking here is that the tessellating species binds PduL and is pulled further into the gel, while the non-tessellating species remains unchanged. Native PduA behaves as usual and shows the usual band shift on the addition of PduL. *D*, confirmation that on the titration of PduL into PduA the mobility of PduA increases. Here His-tagged PduA and tagless PduL are used. The increase in mobility can be seen in the Native-PAGE (*upper gel*) and the anti-His antibody Western blot confirms that PduA has increased mobility in the complex. The binding of PduL dimer to the PduA hexamer saturates at 1:1. We know there are three hexamers of PduA from the results described above, so there must also be three dimers of PduL. The 1:1 stoichiometry of PduL dimer to PduA hexamer can be seen also in Figure 5*A*. Some lanes were cropped from the gel images shown in panels (*B*) and (*C*) at the position indicated by the *dotted lines*.

We explored the binding of PduL to tessellating and mutated non-tessellating variants of PduA. A distinct band shift is observed on the native gel when PduL is titrated into tessellating PduA hexamers but not when added to the non-tessellating mutant, K26D PduA (Fig. 3*A*). As expected, the non-tessellating K26D PduA has higher mobility than the tessellating native PduA because of its lower mass and greater negative charge. The increase in the mobility of PduA with the addition of PduL was not expected. It is plausible that PduL binds to the tessellating hexamers and pulls the complex further into the gel. PduA has a pI of 8.0 and the gel is run at pH 8.5 while PduL with pI of 6.2 confers a negative charge and higher mobility to the complex. Another possibility is that PduL is breaking up the tessellating PduA hexamers while still binding to the individual hexamers. This is a less likely explanation as PduL would bind to both tessellating and non-tessellating forms of PduA. However, it might be argued that the mutation K26D might prevent the binding of PduL to the hexamer. A non-tessellating concatenated PduA, A6, and a slowly tessellating A6 variant were also used to explore this possibility. In the A6 variant, the six chains of the PduA hexamer are concatenated into a single polypeptide chain with linkers between the six concatenated subunits. When PduL is titrated into A6 no complex is formed, and the proteins migrate independently (Fig. 3*B*). This result again links tessellation to PduL-binding. The behavior of the slowly tessellating PduA A6 variant, (K26D)_4_, is interesting (Fig. 3*C*). This variant has four of the six concatenated PduA copies with aspartate in place of lysine 26 and two retain the original lysine. Due to the addition of PduL, the tessellating fraction of the sample undergoes a band-shift, but the non-tessellating component of the sample does not (Fig. 3*C*). This result strongly supports the view that tessellation is needed for PduL binding. It is difficult to argue that PduL binding resulting in the disassembly of tessellation would account for this result. A titration of PduL into PduA reveals a 1:1 binding of PduL dimer to PduA trimer (Fig. 3*D* upper panel). The Western blot (Fig. 3*D* lower panel) using an anti-His antibody to His-tagged PduA (PduL is not tagged and does not bind the antibody) shows PduA is present in the lower band on the native gel, revealing that this band is the complex of PduA and PduL.

### *In silico* modeling of cargo-encapsulation peptide binding

**Figure 4:**
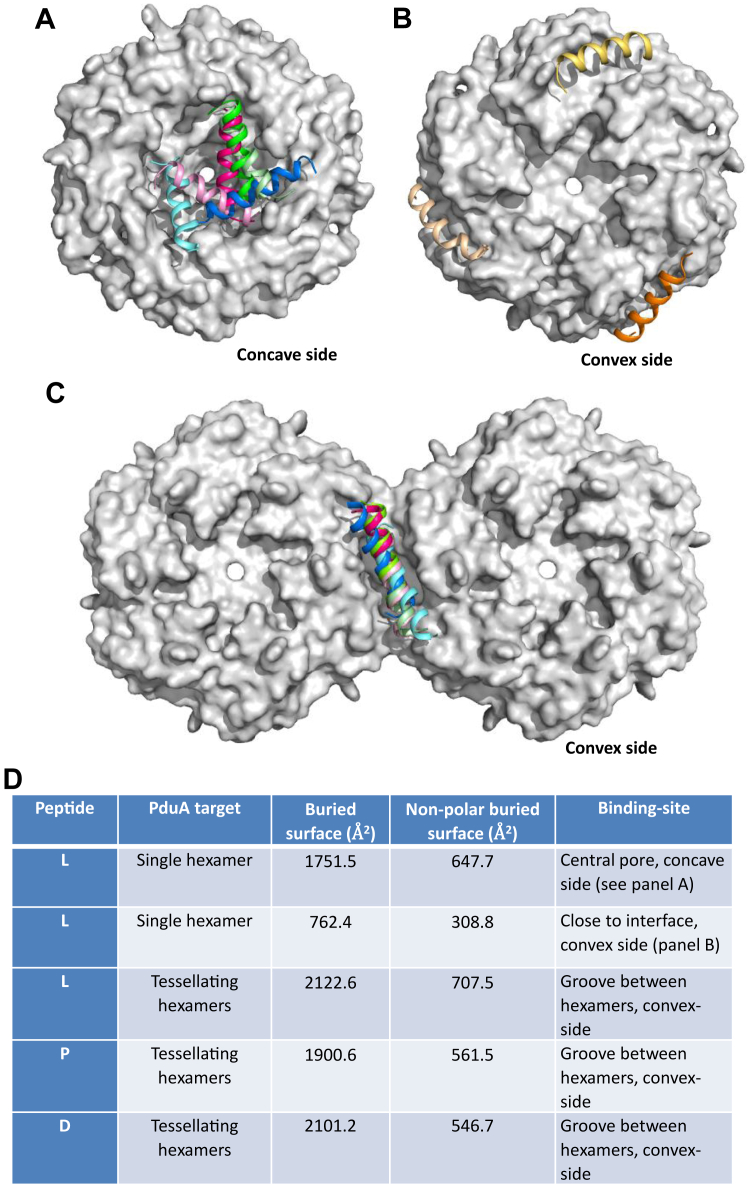
***In silico* modeling of the structure of the encapsulating peptide binding to the PduA hexamer and to tessellating hexamers.***A*, top two hits from docking the encapsulation peptides with the PduA hexamer: L20 (the first 20 residues of PduL in *light and dark pink*), P18 (*light and dark green*), and D18 (*light and dark blue*). The binding is to the concave side of PduA. *B*, the third-ranked hits for all three encapsulating peptides bind to the convex side of the hexamer close to the hexamer–hexamer interface. *C*, when presented with tessellated hexamers, despite given no preferred binding site residues on PduA, all three encapsulation peptides localise to the cleft between hexamers on the convex side of the PduA dimer. *D*, a summary of peptide binding to PduA hexamer and tessellating hexamers.

The binding of the cargo encapsulation peptides of PduL (L20), PduD (D18), and PduP (P18) first to the PduA hexamer and then to the dimer of hexamers was evaluated *in silico* using three docking methods: ClusPro (30), Frodock (31) and CABS-dock server (32). The peptides were modeled both as helical and as flexible peptides and the search covered the entire surface of the hexamer and of the dimer of hexamers. When the surface of the monomer was searched, the peptides bound to the concave side of the PduA disk in the mode described previously (22) (Fig. 4*A*). The second-ranked hit is substantially the same as the top hit, but the third-ranked it is on the convex side close to the hexamer-hexamer interface (Fig. 4*B*). When the surface of tessellating PduA hexamers is searched, the results consistently show binding to the groove at the hexamer-hexamer interface (Fig. 4*C*). For instance, the rmsd for L20 binding to the hexamer-hexamer interface, using CABS-dock, was 0.88 Å with a cluster density of 113 (compared to the significantly poorer values of 3.0 Å and 27 for binding to a single hexamer). In this CABS-DOCK model, the irregular starting peptide structure is predicted to bind to the groove as an amphipathic helix (Fig. 4*C*). In summary, the cargo-encapsulation peptides are predicted to bind to the groove between tessellating hexamers with higher affinity than to individual hexamers (Fig. 4, *C* and *D*).

### Titration of the PduL dimer into the PduA trimer of hexamers

**Figure 5:**
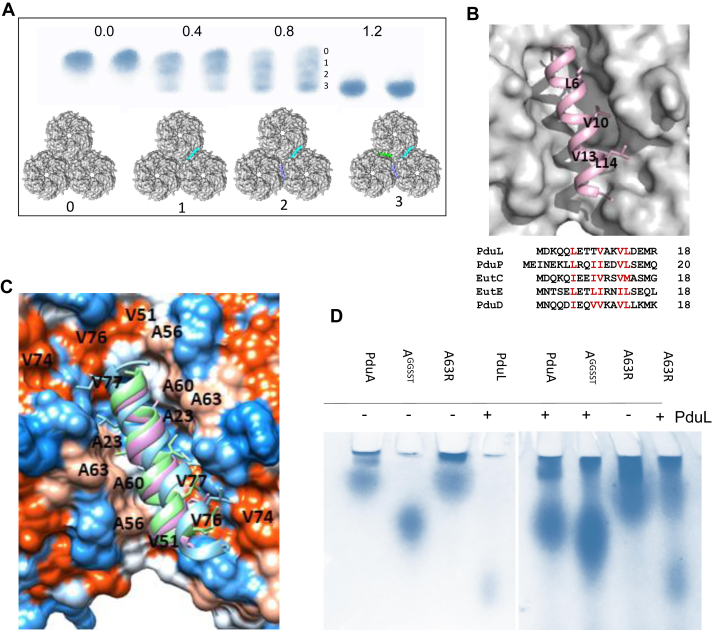
**Further exploration of PduL binding to PduA.***A*, the *top row* is a gradient native gel showing the shift from PduA hexamers (*left* pair of columns; 0.0 PduA) to 1:1 ratio of PduL dimers to PduA trimer of hexamers (*right* pair of columns). The intermediate two pairs of columns show the progress during titration with four species, labelled 0 to 3, clearly seen in the third pair of columns of the panel. These bands correspond to PduA trimer with none (labelled 0), with one (labelled 1), with two (labelled 2) and with three PduL (labelled 3) dimers bound as illustrated bound to the surface of the PduA trimer of hexamers on the *bottom* row of the panel. *B*, closeup of the encapsulation peptide binding site between tessellating hexamers of PduA. The conserved hydrophobics which form a patch on the surface of the helical encapsulation peptides are shown as sticks and labeled. *C*, the position of conserved small hydrophobics on the surface of PduA on α-helix 1 (AMVKSA; residues 23 and 27 underlined) and α-helix 2 (AATDAGAAAA; 56, 60 and 63 underlined). The mutation A63R was designed to block the binding site. *D*, the result of the band-shift assay using the PduA A63R mutant. Both PduA and A63R can be seen to tessellate (low mobility on the gel). The PduA A63R mutant is however unable to bind PduL whereas PduA undergoes the usual band shift. PduA with C-terminal linker, GGSST, is used here as a non-tessellating control (A^GGSST^), it does not bind PduL.

The stoichiometry of binding of 1:1 for the PduL dimer to PduA trimer of hexamers was originally established using the 6% acrylamide gel (Fig. 3*D*). The binding was subsequently explored at higher resolution using a native gradient gel. The lower mobility band on this gel corresponds to the PduA trimer of hexamers and the highest mobility band to the complex of three PduL dimers per PduA trimer of hexamers (Fig. 5*A*). As the ratio of PduL increases across the gel from left to right, two bands are observed between the unbound and fully saturated PduA trimer of hexamers. We interpret the intermediate bands as the binding of one and two PduL dimers per PduA trimer of hexamers (Fig. 5*A*). PduA alone forms nanotubes but in the presence of PduL or other encapsulation peptides, only sheets are seen in electron micrographs (Fig. S1). This supports the view that the cargo-encapsulation peptide is binding between hexamers because when the groove is occupied by peptide the hexamer-hexamer interface cannot bend to the angle required to make a tube (28). The apparent cooperativity of binding is interesting (Fig. 5*A*), and is considered in the discussion, it is consistent with binding at or close to the hexamer-hexamer interface.

### A mutation that allows PduA tessellation but prevents PduL binding

The proposed binding of the encapsulation peptide to the tessellation interface with conserved hydrophobic residues highlighted is shown in Figure 5*B*. The mutation A63R was made to introduce a bulky group to PduA block the PduL binding site identified in the modeling studies (Fig. 5*C*). PduA A63R was seen to form sheets using electron microscopy so tessellation of the PduA hexamers is preserved. This mutant is more basic than PduA so required the native gel to be run at pH 9.5 instead of pH 8.5. Compared to the non-tessellating mutant with the C-terminal extension (GGSST), A63R has similar mobility to PduA confirming that it can successfully tessellate to form a trimer of hexamers (Fig. 5*D*). When PduL is added to A63R no binding is observed with the two bands, PduL and A63R, running as separate bands with unchanged mobility. This contrasts with the band shift seen with native PduA (Fig. 5*D*). This result reveals that if the groove formed between tessellating hexamers is blocked by the mutation A63R then the mutant PduA is unable to bind the PduL encapsulation peptide.

## Discussion

Using gentle sonication to disrupt higher-order assemblies and higher pH to slow subsequent reassembly we have been able to separate early-assembly intermediates in the formation of PduA sheets and nanotubes. Frequently observed, the higher molecular mass assembly of PduA hexamers travels more slowly through the native gel than variants that do not tesselate. Tessellation of PduA was confirmed using electron microscopy and observing the presence of sheets and nanotubes. These higher-order structures were not seen using non-tessellating variants, and nanotubes were not seen when encapsulation peptides were added. The binding of the cargo-encapsulation peptide to the groove between tessellating hexamers will restrict the bending of the hexamer-hexamer interface that is necessary to form tubes. Of the dimer and trimer of hexamers resolved on the native gel, the cyclic trimer with 3-fold symmetry is proposed to be the more stable assembly intermediate because each of the three tiles is stabilized by interaction with two adjacent tiles. The non-tessellating variants of PduA used in this study have mutations near the hexamer-hexamer interface or the addition of flexible linkers that are presumed to interfere with the hexamer-hexamer interface. Unlike tessellating PduA, these non-tessellating variants do not bind PduL. This is either because the cargo-encapsulation peptide binds close to the hexamer-hexamer interface and mutation disrupts the interface or because the encapsulation peptide binds in the hydrophobic groove formed between adjacent tessellating hexamers. In either case, this is a new result and links the interface to encapsulation peptide binding. The correlation of tessellation and cargo-encapsulation peptide binding is striking and established using several mutants of PduA including the slowly tessellating mutant where only the tessellated oligomer binds to PduL.

The experimental results produced by Fan *et.al.* (2014) are in broad agreement with our proposed model. They show that the mutations E7A, I10A, and L14A abolish the incorporation of PduP into microcompartments. In our proposed binding model these latter two hydrophobic residues are buried at the hydrophobic interface between the cargo-encapsulation peptide helix and groove between tessellating tiles. The reason for the conservation of Glu 7 is less clear, but it might interact with Arg 66 of PduA.

*In silico* studies suggest the binding of the cargo-encapsulation peptides is preferentially to the hydrophobic grooves between the tessellating hexamers. The binding of the encapsulation peptide to this convex side of the hexameric disk agrees with previous studies showing this side of the hexamer faces the lumen of the microcompartment. The peptides adopt helical conformation on binding and present a hydrophobic surface to the binding site. This mode of binding would not be readily detected in previous *in silico* studies using isolated hexamers possessing half the binding site. The conserved hydrophobic residues of the helical encapsulation peptides interact with conserved small hydrophobic residues on the α1 and α2-helices of two adjacent PduA hexamers. The residues on the first helix are: 23 and 27; and on the second: 56, 60, and 63. Mutation of one of these hydrophobic residues, Ala 63, to the bulkier and charged arginine, blocks the binding cleft, transforms its hydrophobic character, and prevents the binding of the encapsulation peptide despite allowing tessellation of the PduA hexamers. This mutagenesis result supports the *in silico* modeling and binding of cargo-peptide to the hydrophobic groove between tessellating tiles.

Further evidence for binding between tessellating hexamers comes from the higher-resolution titration which shows saturation at three PduL dimers per PduA trimer consistent with the binding to the three hexamer-hexamer interfaces present in the trimer. In this model, one encapsulation peptide from the PduL dimer interacts with the PduA interface and the other encapsulation peptide is free to interact with another microcompartment component. A plausible explanation for the positive cooperativity seen is that the binding of two encapsulation peptides flattens the assembly of three hexamers, opens the third binding groove, and thereby increases the affinity for the third peptide. Electron microscopy shows that the presence of the encapsulation peptides inhibits the formation of PduA nanotubes which require bending at the junction between hexamers. We can now understand how the encapsulation peptide might influence the assembly of the microcompartment *in vivo*. Binding to a single hexamer would not directly affect assembly, except by increasing the effective concentration in the vicinity of the enzyme but binding between hexamers will stabilize the hexamer–hexamer interface, increase facet stability, planarity, and size, and directly promote microcompartment assembly. The groove between tessellating hexamers has two-fold symmetry, this complicates achieving ordered binding of peptides for experimental structural studies. When the encapsulation peptides are attached to their cargo enzymes, the oligomeric state of the cargo and the steric exclusion of the enzymes will affect how the peptides are presented and it is plausible this will also influence binding to microcompartment facets. PduL is a dimer and the results presented here suggest that one N-terminal encapsulation peptide from the PduL dimer binds to the trimer of PduA hexamers and the other encapsulation peptide is unbound.

A prominent hydrophobic groove formed between tessellating tiles is a common theme in the crystal structures of shell proteins and the sequence of small hydrophobics of α-helix 1 (AMVKAA; residues 23 and 27 underlined) and α-helix 2 (AATDAGAAAA; 56, 60 and 63 underlined, 3NGK numbering) that form the binding-site is conserved across many hexameric shell proteins including the major shell proteins PduJ and PduA (Fig. S2). This hydrophobic groove is therefore present in the facets of the bacterial microcompartment shell. We suggest that encapsulation peptide binding to grooves formed between tessellating subunits is a general way of binding cargo and plausibly also of prompting nucleation of the microcompartment shell.

## Experimental procedures

### Molecular biology

We previously described a variant of *C. freundii* PduA with a C-terminal 23 residue extension which aids protein solubility (15). This is the variant used in this work, it is referred to in the methods as PduA∗, but simply as PduA in the main text. The concatenated constructs of 6 sequential copies of PduA∗ were prepared in pOPIN F (OPPF) and modified to contain a TEV cleavage site and the restriction sites *SpeI*, *EcoRV*, and *BglII* inserted into the *KpnI* site. PduA∗ or PduA∗_K26D_ were amplified using primers containing *ScaI* in the forward primer (5′-3′) ggagtactatgcaacaagaagcgttagg and incorporating *EcoRV* and *BglII* sites either side of a stop codon using the primers (5′-3′) atagatctttagatatcttgctcagcggtggcagc. The PCR product was ligated into pBluescript SKII +. The gene was excised using *ScaI* and *BglII* and was ligated into pOPIN F TEV linearised with *EcoRV* and *BglII*. This was repeated in a link and lock style (33) approach until 6 copies of PduA, with the 23-residue extension between each repeat, had been ligated. PduA_GGSST_ was designed to include a 30 residue C-terminal extension (six repeats of GGSST), in place of the C-terminal 23 residue extension tag and was synthesized including the same *ScaI*, *EcoRV*, and *BglII* sites as above. The synthesized gene was excised from the commercial vector pEXA128 (Genewiz) using *ScaI* and *BglII* and ligated into pOPIN F (OPPF) containing a TEV cleavage site linearised with *EcoRV* and *BglII*. PduA∗ A63R was created using site-directed mutagenesis. All sequences were confirmed by sequencing (Source BioSciences).

The gene for *C. freundii* PduL was originally cloned into pET3a (modified with *SpeI* 5′ of *BamHI*). The forward primer with *NdeI* restriction site used was gcgcatatggataaacagcaactggag and the reverse with *SpeI* restriction site gcgactagtcatcgtgggctcaccagtg. This was subsequently subcloned into pET28a by an *NdeI* and *BamHI* digest, therefore creating a thrombin cleavable N-terminal His-tag. Again, all were sequences confirmed before protein production.

### Protein production and purification

For protein production, BL21 (DE3) transformed with the desired plasmid were grown in 1 L volumes of 2YT media supplemented with ampicillin, at 37 °C while shaking at 200 rpm. Gene expression was induced at an OD of 1.0 with 0.4 mM IPTG followed by overnight incubation at 18 °C, shaking at 200 rpm. Cells were harvested by centrifugation at 6000*g* for 10 min and were resuspended in 20 mM Tris pH 8.0, 500 mM NaCl. The cells were lysed by sonication and the lysate was clarified using centrifugation at 25,000*g* for 30 min. Proteins were purified using immobilized nickel affinity chromatography. PduA and mutants were washed with 20 mM Tris pH 8.0, 500 mM NaCl, and imidazole up to 150 mM before elution with 500 mM. PduL was washed with 20 mM Tris pH 8.0, 500 mM NaCl, and imidazole up to 60 mM before elution with 250 mM. The N-terminal His-tag of PduL was cleaved after incubation with thrombin at 4 °C overnight. Thrombin and un-cleaved protein were removed using reverse immobilized nickel affinity chromatography. The proteins were further purified using size exclusion chromatography on a Superdex 200 10/300 column equilibrated in 25 mM HEPES, 500 mM NaCl, pH 8.0, and eluted at their expected sizes.

### Native-PAGE analysis and Western blot

For all Native-PAGE, BioRad Mini-PROTEAN TGX 4 to 15% gradient gels and running buffer (25 mM TRIS, 192 mM glycine) were run at 4 °C for 3 h at 100 V fixed with variable current. Native-PAGE samples were prepared in 200 mM NaCl, 25 mM HEPES, pH 8.0, and protein complexes were left at 4 °C for 1 h before the addition of loading buffer (0.1% bromophenol blue, 50% glycerol, 50% 1x running buffer). When using Native-PAGE titration of PduL into PduA, it was necessary to demonstrate the location of PduA. The N-terminal His-tag of PduL was cleaved to both facilitate binding to PduA and enable the exclusive detection of PduA with an anti-His antibody obtained from Novagen. The protein concentrations used were circa 1.0 mg/ml PduA for Native-PAGE.

### Cross-linking

To prepare PduA∗ oligomers, 4 mg/ml PduA∗ in 20 mM HEPES, 500 mM NaCl, pH 8.0, was sonicated, on ice, for 30 s pulses for 2 min. Glutaraldehyde to 1% v/v was added immediately after sonication and the crosslinking reaction was incubated at 4 °C overnight. The reaction was terminated using size exclusion chromatography with Superose 6 column in 20 mM Tris, 500 mM NaCl pH 8.0.

### Dynamic light scattering and mass-spectrometry

Dynamic light scattering of PduA∗ samples was measured using the Protein Solutions DynaPro MS/X. The 4 mg/ml samples were first filtered through a 1 μm filter to remove larger particulates. MALDI-TOF mass-spectrometry of PduA∗ was using a Bruker rapifleX MALDI PharmaPulse spectrometer. Sinapinic acid was added in excess to 30% water, 70% acetonitrile, 0.1% TFA. This mixture was then vortexed thoroughly and centrifuged for 1 min at 20,000 RPM. The supernatant (saturated with sinapinic acid) was removed and added to 1 mg/ml of desalted protein in a 1:1 ratio. 1 μl of this mixture was added to the sample plate and left until thoroughly dried. Sample analysis then proceeded using positive detection mode, collecting 1000 images per sample.

### Structural modeling

Models of a PduA∗ hexamer and a pair of hexamers were produced using the structure 3NGK (14). ClusPro (30), Frodock (31), and CABS-dock server (32) were then run on the hexamer and dimer of hexamers using the peptides L20, P18, or D18. CABS-dock simulation was run for 50 cycles and no preferred regions were selected to avoid any implicit bias. All structural figures presented were made using PyMOL (34).

## Data availability

All data are contained within the manuscript.

## Supporting information

This article contains supporting information.

## Conflict of interest

The authors declare that they have no conflicts of interest with the contents of this article.
