# Supplementary material for: Enzyme-cargo encapsulation peptides bind between tessellating tiles of the bacterial microcompartment shell
Source: J Biol Chem. 2024 May 10;300(6):107357. doi: 10.1016/j.jbc.2024.107357 (PMC11157265; doi:10.1016/j.jbc.2024.107357)
Supplement: Supporting Information [file mmc1.docx]

**Supplementary Information for:**

**Enzyme encapsulation peptides bind tessellating tiles of the bacterial microcompartment shell**

Shuang Gu, Jack Bradley-Clarke, Ruth Rose, Martin J. Warren, Richard W. Pickersgill

Corresponding author: Richard W. Pickersgill

Email: [r.w.pickersgill@qmul.ac.uk](mailto:r.w.pickersgill@qmul.ac.uk)

**Table of contents**

**Figure S1**. Evidence from electron microscopy that the presence of encapsulation peptides or enzymes with encapsulation peptides prevents curvature of PduA sheets.

**Figure S2**. Conservation of the encapsulation peptide binding site in PduA, PduJ and EtuM.


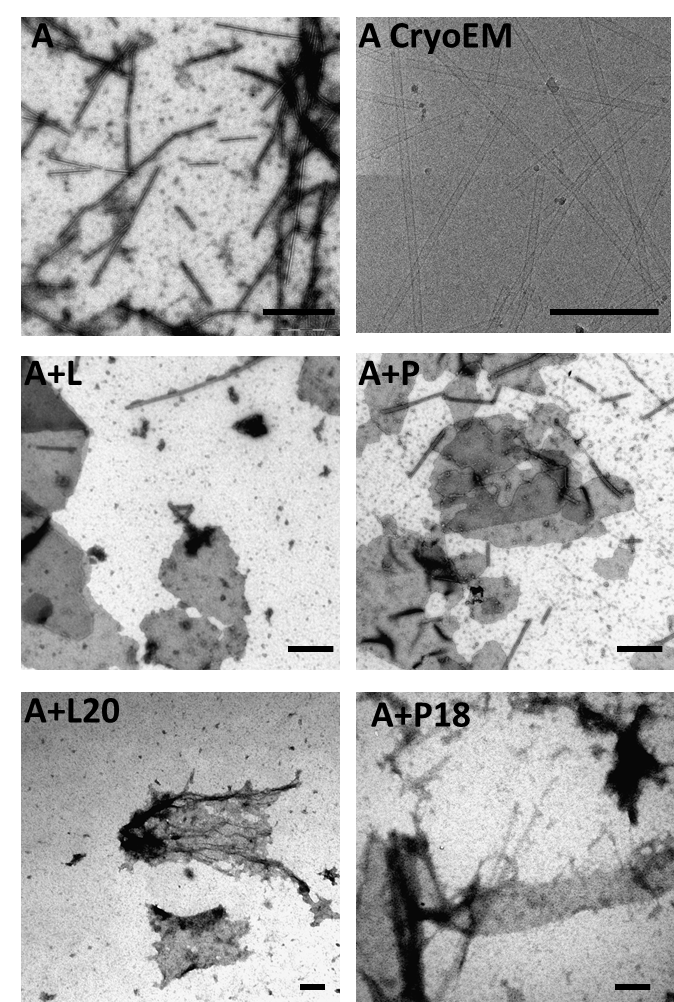


**Figure S1**. Evidence from electron microscopy that the presence of encapsulation peptides or enzymes with encapsulation peptides prevents curvature of PduA sheets. The top two panels show PduA alone in negative stain and in cryo conditions; the lower four panels show PduA in the presence of the enzymes PduL and PduP and their targeting peptides L20 and P18. PduA alone predominantly forms nanotubes, but in the presence of enzyme or peptide nanotubes are not favoured and sheets are dominant. This is consistent with encapsulation peptide binding at or near the hexamer-hexamer interface. The scale bar shown is 200nm.


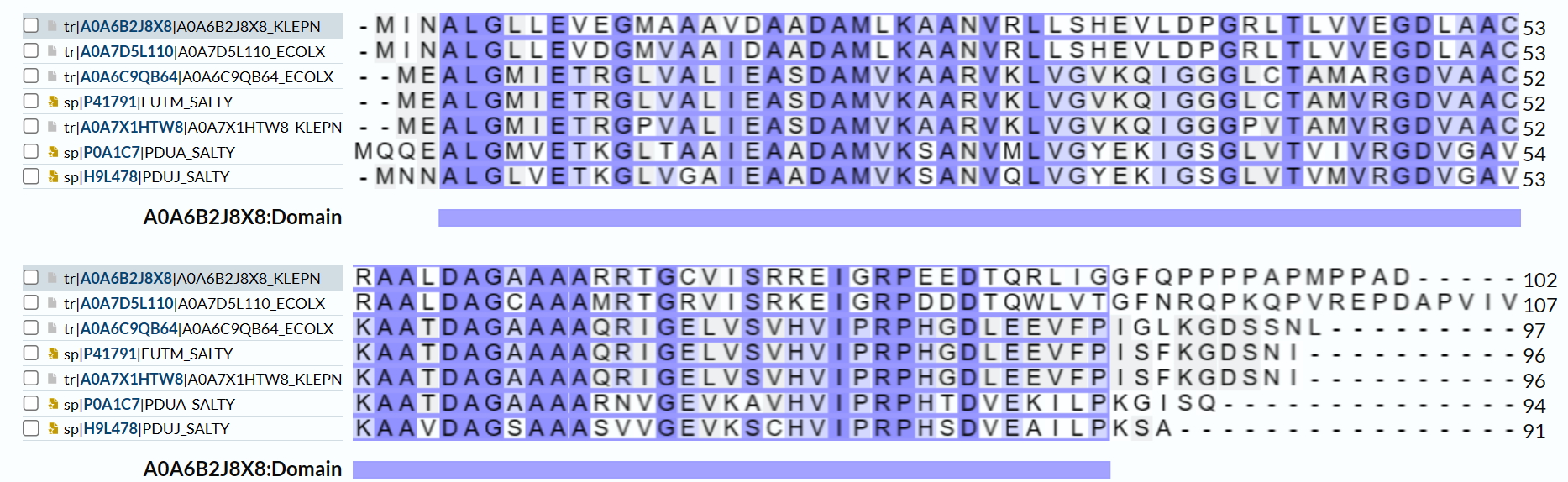


**Figure S2**. Conservation of the encapsulation peptide binding site in shell proteins PduA, PduJ and EtuM. PduJ and PduA together account for over 70% of the shell proteins of the *Salmonella typhimurium* LT2 Pdu microcompartment. The conserved residues forming the encapsulation binding site on PduA are conserved in the major shell protein PduJ (see the conservation of binding site in the red boxes). The two signature sequences are on the surface of alpha-helices 1 and 2: AADA^23^MVK(A/S)^27^A and A^56^ATDA^60^GAA^63^AA, respectively (PduA, *Salmonella typhimurium* LT2, SALTY, numbering). The PduJ-PduJ interface will be most common and has the same hydrophobic groove as the PduA-PduA interface. Sequences shown in this figure are from *Salmonella typhimurium* LT2 (SALTY), *Klebsiella pneumoniae* (KLEPN), and *E. coli* (ECOLX).
